# Chitosan-Induced Activation of the Antioxidant Defense System Counteracts the Adverse Effects of Salinity in Durum Wheat

**DOI:** 10.3390/plants10071365

**Published:** 2021-07-03

**Authors:** Filippo Quitadamo, Vanessa De Simone, Romina Beleggia, Daniela Trono

**Affiliations:** Consiglio per la Ricerca in Agricoltura e l’Analisi dell’Economia Agraria, Centro di Ricerca Cerealicoltura e, Colture Industriali, S.S. 673, Km 25,200, 71122 Foggia, Italy; filippoquitadamo@libero.it (F.Q.); vanessa.desimone@crea.gov.it (V.D.S.); romina.beleggia@crea.gov.it (R.B.)

**Keywords:** antioxidant enzymes, chitosan, durum wheat, malondialdehyde content, salt stress, total antioxidant activity

## Abstract

The present study was carried out with the aim of (*i*) evaluating the effect of chitosan (CTS) on the growth of durum wheat under salinity and (*ii*) examining CTS-regulated mechanisms of salinity tolerance associated with the antioxidant defense system. To achieve these goals, durum wheat seedlings were treated with CTS at different molecular weight, low (L-CTS, 50–190 kDa), medium (M-CTS, 190–310 kDa) and high (H-CTS, 310–375 kDa). The results obtained show that exposure to 200 mM NaCl reduced the shoot and the root dried biomass by 38% and 59%, respectively. The growth impairment induced by salinity was strongly correlated with an increase in the superoxide anion production (5-fold), hydrogen peroxide content (2-fold) and malondialdehyde (MDA) content (4-fold). Seedlings responded to the oxidative stress triggered by salinity with an increase in the total phenolic content (TPC), total flavonoid content (TFC) and total antioxidant activity (TAA) by 67%, 51% and 32%, respectively. A salt-induced increase in the activity of the antioxidant enzymes superoxide dismutase and catalase (CAT) of 89% and 86%, respectively, was also observed. Treatment of salt-stressed seedlings with exogenous CTS significantly promoted seedling growth, with the strongest effects observed for L-CTS and M-CTS, which increased the shoot biomass of stressed seedlings by 32% and 44%, respectively, whereas the root dried biomass increased by 87% and 64%, respectively. L-CTS and M-CTS treatments also decreased the superoxide anion production (57% and 59%, respectively), the hydrogen peroxide content (35% and 38%, respectively) and the MDA content (48% and 56%, respectively) and increased the TPC (23% and 14%, respectively), the TFC (19% and 10%, respectively), the TAA (up to 10% and 7%, respectively) and the CAT activity (29% and 20%, respectively). Overall, our findings indicate that CTS exerts its protective role against the oxidative damages induced by salinity by enhancing the antioxidant defense system. L-CTS and M-CTS were the most effective in alleviating the adverse effect of NaCl, thus demonstrating that the CTS action is strictly related to its molecular weight.

## 1. Introduction

Durum wheat (*Triticum durum* Desf.) is one of the most important cereal crop species in terms of human consumption in the countries of the Mediterranean basin, where it is extensively cultivated [1]. In these coastal areas, salt accumulation in the soil occurs as a consequence of the intrusion of seawater into aquifers and the irrigation through salty water [2,3]. This problem is becoming more serious due to the effect of climate change, that is expected to further exacerbate seawater intrusion due to sea-level rise [4]. Salt accumulation leads to permanent modifications of the physical–chemical properties of the soil [5] and reduces the normal plant growth and productivity of agricultural crops [6,7]. Durum wheat is a cereal species well adapted to rainfed environments, but, compared to bread wheat, this crop is generally less tolerant to salinity. Consequently, durum wheat yield and quality may be substantially affected by high salt levels [8,9]. In the light of this, understanding the mechanisms that underlie salinity tolerance in durum wheat represents a valuable approach to develop new salt-tolerant, high-yielding genotypes.

Salt stress exerts its negative effects on plant physiology and metabolism by inducing osmotic stress and ion imbalance and toxicity [10,11]. Moreover, salinity, like other abiotic stresses, leads to an increase in the formation of reactive oxygen species (ROS), such as superoxide anion (O_2_^•−^), hydrogen peroxide (H_2_O_2_), hydroxyl radical (OH^•^) and singlet oxygen (^1^O_2_), which trigger the oxidation of macromolecules, thus leading to the impairment of cellular integrity and functionality [12]. Among macromolecules, membrane lipids represent an important target of ROS-induced damages and malondialdehyde (MDA), the primary end-product of the membrane lipid peroxidation, is widely used as marker for the estimation of the oxidative damage of biomembranes [13]. To maintain stable ROS levels in cell compartments, plants have evolved an antioxidant system composed of both non-enzymatic and enzymatic antioxidants [14,15]. The non-enzymatic antioxidants in plants consist of small molecules that react directly with ROS and scavenge them; these include, among others, phenolic compounds, tocols, glutathione and ascorbate [16]. Phenolic compounds have shown a greater antioxidant activity compared to other small antioxidant molecules [17]. These molecules are very suitable for trapping free radicals and this ability is related to their structure that rapidly stabilizes the formed phenol radical; in particular, phenolic compounds efficiently inhibit lipid peroxidation by trapping lipid alkoxy radicals [18]. The antioxidant enzymes comprise the superoxide dismutase (SOD), which catalyzes the dismutation of the superoxide anion to oxygen and hydrogen peroxide, and the catalase (CAT) and the peroxidase (POX), which catalyze the decomposition of the hydrogen peroxide into oxygen and water [19]. The hydrogen peroxide is also decomposed by the ascorbate-glutathione cycle, which uses small non-enzymatic antioxidants and NADPH as sources of reducing equivalents [20]. The principal enzymes capable of regenerating the NADPH are the glucose-6-phosphate dehydrogenase (G6PDH) and the 6-phosphogluconate dehydrogenase (6PGDH) (both involved in the pentose phosphate pathway), the NADP-dependent malic enzyme (NADP-ME) and the isocitrate dehydrogenase (ICDH) [21,22]. Evidence has been reported that the dehydrogenase-catalyzed recycling of NADPH plays a key role in counteracting the stress-triggered ROS accumulation [22,23]. Therefore, NADPH dehydrogenases may be considered essential components of the antioxidant defense system. 

One of the recent strategies to improve stress tolerance of crops is the application of naturally occurring molecules, which act as elicitors by triggering stress responses linked to plant defense machinery. Among these compounds, chitosan (CTS) and its derivatives deserve particular attention, because evidence exists about their ability to counteract the detrimental effects of both biotic and abiotic stresses and improve yield and quality of crops [24]. In particular, CTS and CTS-derivatives were found to possess antifungal, antibacterial and antiviral activity and different CTS-based formulations were used, as an alternative to chemical pesticides, to achieve the control of plant diseases [25]. CTS and its derivatives were also found to confer resistance against abiotic stresses, such as drought, salinity, high temperatures and heavy metals [26]. Under both biotic and abiotic stresses, it was reported that the beneficial effects of CTS depended on its structure features, such as the molecular weight (MW) and the degree of deacetylation [27,28].

In the light of the economic importance of durum wheat production, the present study was carried out with the aim of evaluating the efficacy of foliar application of CTS with different MWs, low (L-CTS, 50–190 kDa), medium (M-CTS, 190–310 kDa) and high (H-CTS, 310–375 kDa), in improving salinity tolerance of durum wheat seedlings and examining CTS-regulated mechanisms of salt-stress tolerance associated with the enzymatic and non-enzymatic antioxidant system and the control of the redox state.

## 2. Results

### 2.1. Effect of CTS on the Growth Performance of NaCl-Treated Seedlings

Variations in the growth parameters of durum wheat seedlings were studied to evaluate the damages induced by NaCl stress and to assess the possible protective role of CTS treatments against salinity. As shown in Table 1, salt stress had significant injurious effects on plant growth, more evident in the root than in the shoot. A decrease of 36% and 38% was observed for the shoot length and dried biomass, respectively, whereas the primary root length and root dried biomass decreased by 60% and 59%, respectively. 

The exogenous application of CTS at three different MWs significantly reduced the deleterious effects of NaCl and increased the growth parameters, compared to the stressed seedlings. The strongest effect was observed in stressed seedlings treated with L-CTS and M-CTS. In particular, L-CTS treatment increased the shoot length and dried biomass of stressed seedlings by 30% and 32%, respectively, whereas an increase of 36% and 44%, respectively, was observed after M-CTS treatment (Table 1). An even higher effect was observed in the primary root length and root dried biomass, which increased by 80% and 87%, respectively, after treatment of the stressed seedlings with L-CTS, whereas an increase of 92% and 64%, respectively, was observed after treatment with M-CTS (Table 1). A lower but significant increase in the growth parameters was observed in stressed seedlings treated with H-CTS, also, in this case, more evident for the root (42–54%) than the shoot (10–11%) (Table 1). 

### 2.2. Effect of CTS on ROS Production and MDA Content in the Shoot of NaCl-Treated Seedlings

An experiment was carried out to evaluate the oxidative stress in the form of ROS and MDA accumulation in the shoot of durum wheat seedlings grown under NaCl stress, in the absence and in the presence of foliar treatment with CTS at different MWs. 

**Figure 1:**
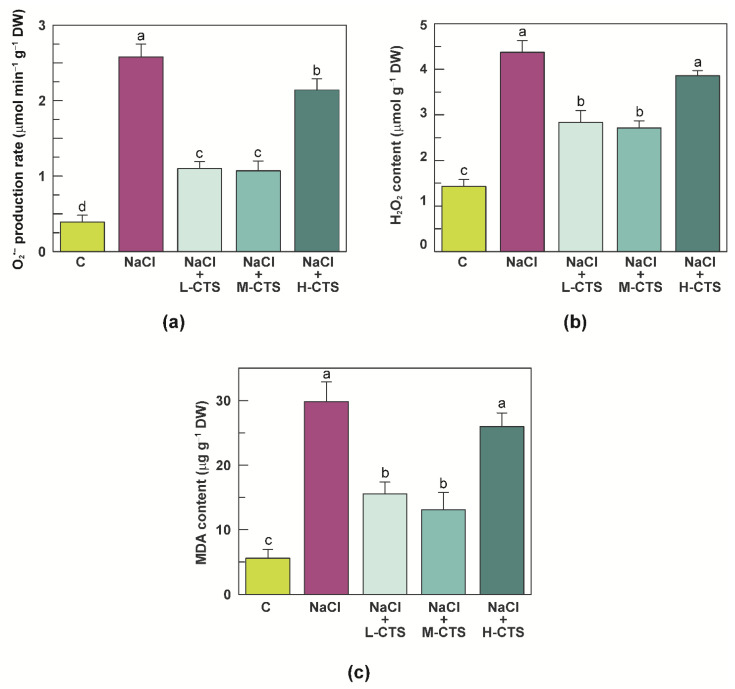
(**a**) Superoxide anion (O_2_^•−^) production, (**b**) hydrogen peroxide (H_2_O_2_) and (**c**) malondialdehyde (MDA) content in the shoot of durum wheat seedlings grown under control, NaCl stress and NaCl stress + CTS treatment. Vertical bars represent S.D. (*n* = 3). Different letters indicate significant differences according to the Tukey’s test (*p* ≤ 0.05).

When seedlings were grown under NaCl stress, a strong increase was observed in the superoxide anion production, hydrogen peroxide and MDA content in the shoot, which reached values more than 5-fold, 2-fold and 4-fold higher, respectively, than the control shoot (Figure 1a–c). 

As already observed for the growth parameters, treatment of stressed seedlings with L-CTS and M-CTS significantly affected both ROS and MDA accumulation. In particular, L-CTS treatment decreased superoxide anion production, hydrogen peroxide and MDA content of stressed seedlings by 57%, 35% and 48%, respectively, whereas a decrease of 59%, 38% and 56%, respectively, was observed after treatment of stressed seedlings with M-CTS (Figure 1a–c). Conversely, no significant effects were observed after treatment of stressed seedlings with H-CTS, except for a slight decrease (17%) in the superoxide anion production (Figure 1a–c).

### 2.3. Effect of CTS on Total Phenolic Content, Total Flavonoid Content and Total Antioxidant Activity in the Shoot of NaCl-Treated Seedlings

The results obtained in the present study show a 67% and 51% increase in the total phenolic content (TPC) and total flavonoid content (TFC), respectively, in the shoot of durum wheat seedlings exposed to salinity (Table 2). Consistently, under stress, a significant increase in the total antioxidant activity (TAA) was also observed to be, on average, 24% and 32% for the DPPH and the ABTS radical scavenging activity, respectively (Table 2).

CTS treatments of stressed seedlings significantly affected TPC, TFC and TAA. In particular, the strongest effect was observed after treatment of stressed seedlings with L-CTS and M-CTS, that increased TPC by 23% and 14%, respectively, whereas TFC increased by 19% and 10%, respectively (Table 2). A less evident effect was observed after treatment of stressed seedlings with H-CTS, that increased TPC and TFC on average by 9% and 7%, respectively (Table 2). In agreement with these results, a significantly higher TAA was observed in the shoot of stressed seedlings after treatment with L-CTS and M-CTS, that increased the DDPH radical scavenging activity by 10% and 7%, respectively, whereas the ABTS radical scavenging activity increased by 6% and 2%, respectively (Table 2). After treatment of stressed seedlings with H-CTS only a slight increase (5%) in the DPPH radical scavenging activity was observed (Table 2). 

### 2.4. Effect of CTS on the Activities of the Antioxidant Enzymes in the Shoot of NaCl-Treated Seedlings

**Figure 2:**
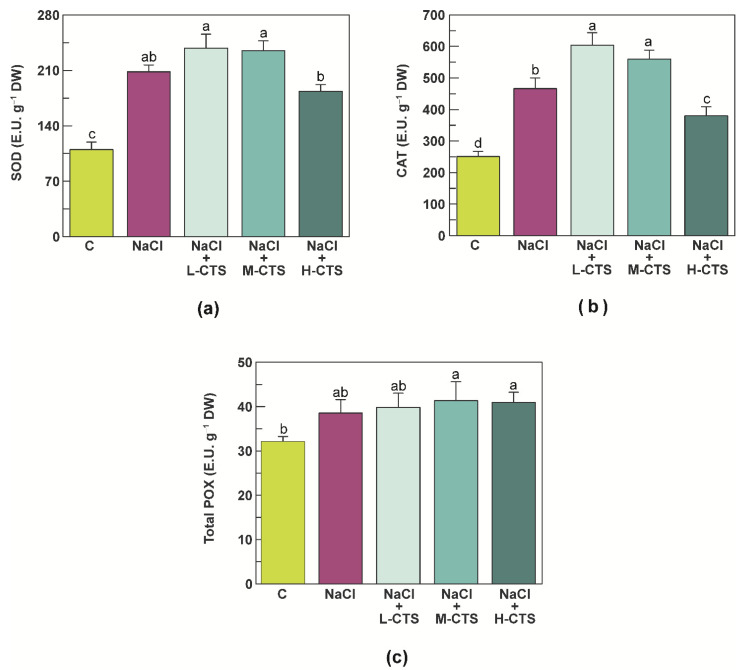
(**a**) Superoxide dismutase (SOD), (**b**) catalase (CAT) and (**c**) total peroxidase (POX) activities in the shoot of durum wheat seedlings grown under control, NaCl stress and NaCl stress + CTS treatment. Vertical bars represent S.D. (*n* = 3). Different letters indicate significant differences according to the Tukey’s test (*p* ≤ 0.05).

In this study, exposure of durum wheat seedlings to NaCl stress induced a significant increase in the activities of the antioxidant enzymes in the shoot. The highest variation was observed for SOD and CAT activities, that increased by 89% and 86%, respectively, compared to control seedlings (Figure 2a,b), whereas only a slight increase (14%) was observed for total POX activity (Figure 2c). 

Foliar treatment of stressed seedlings with CTS significantly affected CAT activity (Figure 2b). L-CTS treatment increased CAT activity by 29%, whereas an increase of 20% was observed after treatment with M-CTS (Figure 2b). Conversely, treatment of stressed seedlings with H-CTS determined a decrease in CAT activity by 19%, although the value remained significantly higher compared to control seedlings (Figure 2b). Only slight variations were observed for SOD and total POX activity after treatment of stressed seedlings with CTS (Figure 2a,c).

### 2.5. Effect of CTS on the Activities of NADPH-Producing Enzymes in the Shoot of NaCl-Treated Seedlings

**Figure 3:**
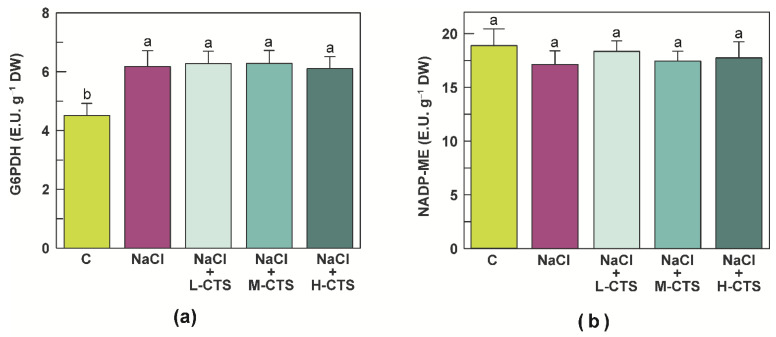
(**a**) Glucose-6-phosphate dehydrogenase (G6PDH) and (**b**) NADP-dependent malic enzyme (NADP-ME) activities in the shoot of durum wheat seedlings grown under control, NaCl stress and NaCl stress + CTS treatment. Vertical bars represent S.D. (*n* = 3). Different letters indicate significant differences according to the Tukey’s test (*p* ≤ 0.05).

An experiment was carried out to evaluate the effect of NaCl exposure of durum wheat seedlings on the activity of two main NADPH-producing enzymes, the G6PDH and the NADP-ME, and to assess the possible interaction between salinity and CTS treatment. Salt stress induced a significant increase (37%) in the activity of the G6PDH (Figure 3a), whereas the NADP-ME was found to be unaffected by salinity (Figure 3b). Compared to stressed plants, none of the CTS treatments resulted in significant changes in either the G6PDH or the NADP-ME activity (Figure 3a,b).

### 2.6. Correlation Matrix among Traits

**Figure 4:**
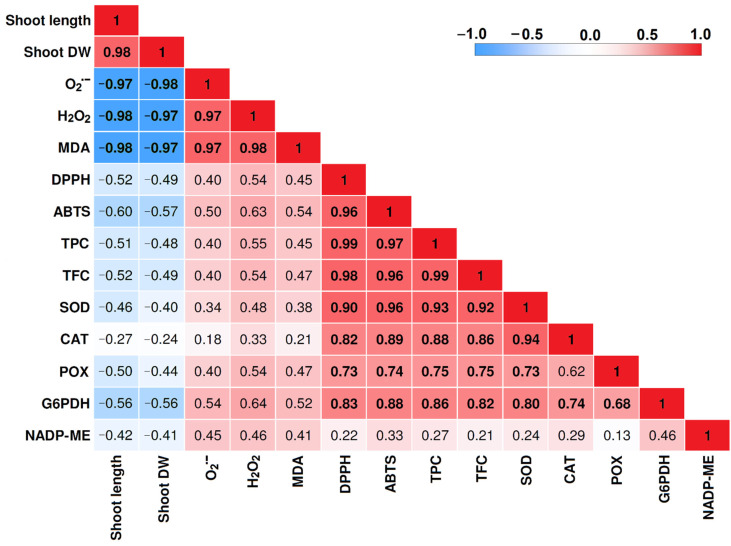
Correlation matrix for the traits analyzed in the shoot of durum wheat seedlings. Red indicates positive correlation and blue indicates negative correlation. Correlations significant at *p* ≤ 0.01 are highlighted in bold.

The correlation matrix for the traits examined in the shoot of durum wheat seedlings is reported in Figure 4. A positive correlation was observed between shoot length and shoot DW (r = 0.98). A positive correlation was also found between superoxide anion and hydrogen peroxide production (r = 0.97) and between the accumulation of these two ROS species and the accumulation of MDA (r = 0.97–0.98). Interestingly, the accumulation of superoxide anion, hydrogen peroxide and MDA were negatively correlated with shoot length and shoot DW (r = −0.97–−0.98). 

A positive correlation was observed between TPC and TFC (r = 0.99), among all the enzyme activities (r = 0.62–0.94), except NADP-ME activity (which did not correlate with any of the traits investigated), and between the content of both phenolic and flavonoid compounds and the enzymatic activities (r = 0.75–0.93). Finally, DPPH and ABTS radical scavenging activities were found to be positively correlated to each other (r = 0.96) and both were positively correlated with TPC and TFC (0.96–0.99) and with the enzymatic activities (r = 0.73–0.96).

### 2.7. Principal Component Analysis

**Figure 5:**
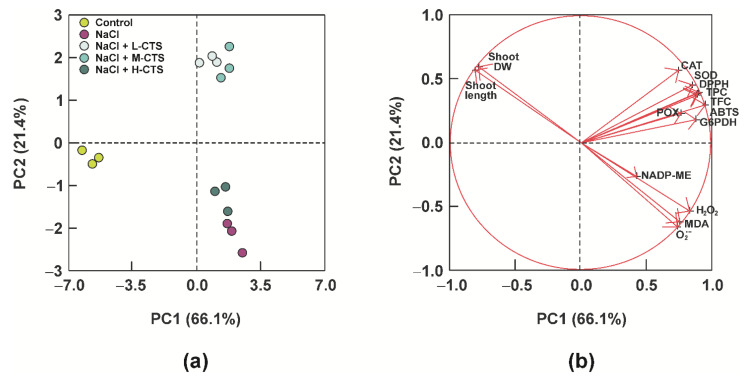
Principal component analysis (**a**) score plot and (**b**) loading plot for the traits analyzed in the shoot of durum wheat seedlings as function of the different treatments. The percentages of total variance represented by principal component 1 (PC1) and principal component 2 (PC2) are shown in parentheses. In (**a**), colored symbols correspond to the five treatments imposed on durum wheat seedlings. In (**b**), vectors indicate the direction and strength of each variable to the overall distribution.

To evaluate the relationships among the growth parameters, the redox state and the components of the antioxidant system of durum wheat seedlings in response to salt stress and CTS treatments, the whole dataset obtained for the shoot was subjected to principal component analysis (PCA). The first three principal components (PCs) represented more than 93% of the variability in the dataset, with PC1 and PC2 that explained 66.1% and 21.4% of the total variance, respectively. The score plot identified three well-defined clusters (Figure 5a). In particular, PC1 separated the effect of NaCl and CTS-treatments from the control, whereas L-CTS and M-CTS treatments were well differentiated from salt stress and H-CTS treatment on PC2 (Figure 5a). As shown in the loading plot (Figure 5b), PC1 was positively correlated with all the variables, except the shoot length and the shoot DW, which were negatively correlated with PC1. PC2 was negatively correlated with superoxide anion, hydrogen peroxide and MDA content and positively correlated with all the other variables. 

## 3. Discussion

In the last decade, an increasing number of researchers have investigated the effects of CTS-based compounds on plants. Several studies have demonstrated the promoting effect of CTS on plant growth and evidence has been reported that this property is linked to the ability of CTS to enhance plant resistance against pathogens [25] and to alleviate the adverse effect of abiotic stresses [26]. Based on the previous studies, the present work investigated the effect of foliar spray application of CTS at three different MWs (L-CTS, M-CTS and H-CTS) on durum wheat seedlings exposed to 200 mM NaCl to assess the possible role of this biopolymer in mitigating the adverse effect of salinity.

Durum wheat is considered a moderately tolerant crop, whose growth and productivity are inhibited at high salt concentrations [29]. Consistently, we previously observed that, when durum wheat seedlings were exposed to different NaCl concentrations (from 50 mM to 200 mM), the detrimental effects on plant growth were observed only at the highest salinity level [9]. In light of this, 200 mM NaCl was chosen for this study. The results obtained in the present study confirm that the salt concentration applied was sufficiently high to impair the shoot and root growth of durum wheat seedlings. As already observed in our previous work [9], growth was more inhibited in the root than the shoot and this was probably due to the osmotic stress suffered by the root, which was directly in contact with the NaCl solution [10]. Foliar application of CTS alleviated the deleterious effect of salt stress on seedling growth. This result confirms the capability of CTS in ameliorating the adverse effects of salt stress already observed in other plant species including dicot [30,31] and monocot [32,33]. In this study, the best effects were observed in durum wheat seedlings treated with L-CTS and M-CTS, whereas H-CTS had the lowest effect. Consistently, Moolphuerk and Pattanagul [34] have recently reported that low MW CTS was the most effective in promoting root growth in rice seedlings exposed to drought, followed by medium and high MW CTS. Similarly, Zong and coworkers [35] have found that foliar application of CTS promoted the plant growth in edible rape leaves exposed to cadmium stress and that the stimulating effect of CTS was dependent on its MW, with low MW CTS showing the highest activity. Low MW CTS has been also demonstrated to be the most effective in promoting growth of tomato seedlings exposed to biotic stresses [36].

In the present study, the shoot of durum wheat seedlings exposed to salt stress increased the accumulation of superoxide anion, hydrogen peroxide and MDA. The positive correlation found between ROS production and MDA accumulation indicated that the increased lipid peroxidation observed under salinity was a direct effect of ROS on polyunsaturated components of membranes. The excessive accumulation of superoxide anion, hydrogen peroxide and MDA are common responses to salinity already observed in many other plant species, such as in *Linum usitatissimum* L. [37] and Indian mustard [38] leaves, cotton [39] and lentil [40] seedlings and mung bean roots and leaves [41]. Our results showed that the application of CTS at low and medium MW to durum wheat seedlings exposed to salinity was able to effectively reduce the accumulation of ROS and, consequently, ameliorate the stability of the membranes, as demonstrated by the decrease induced in the MDA content. The observation that ROS production and MDA accumulation were negatively correlated with shoot growth indicated that the growth impairment observed in durum wheat seedlings exposed to salinity was the consequence of the oxidative stress induced at cellular level by salinity and suggested that the effectiveness of CTS as growth stimulator might be linked to its ability to reduce the ROS levels inside the cell and restore a balanced redox state. To verify this assumption, we evaluated the existence of CTS-regulated mechanisms of salinity tolerance associated with the non-enzymatic and enzymatic components of the antioxidant system. 

Phenolic compounds, including flavonoids, represent one of the most abundant families of secondary metabolites in plants that are produced by the phenylpropanoid pathway and are involved in key metabolic and physiological processes in plants, including the adaptation processes under stress conditions [42]. Evidence exists that the phenylpropanoid biosynthetic pathway is activated under abiotic stresses and this results in the accumulation of various phenolic compounds that are capable of scavenging free radicals, thus resulting in a reduction of cell membrane peroxidation and protection of plant cells from the deleterious effects of the oxidative stress [42]. Our result showed a significant increase in TPC and TFC in the shoot of durum wheat seedlings exposed to salt stress accompanied by a concomitant increase in the TAA. This observation agrees with data already reported in the literature. A salt-induced increase in the total phenolic compounds accompanied by a concomitant increase in the total antioxidant activity has been also observed in lettuce [43], artichoke [44] and maize [45]. In this latter case, the authors reported that the accumulation of phenolic compounds and the increase in the antioxidant activity were more evident in the salt-tolerant compared to the salt-sensitive genotype and speculated that this result could explain why the salt-tolerant genotype had the lowest ROS production and lipid peroxidation [45]. Under salt stress, an increase in the synthesis of phenolic compounds was also observed in *Salvia mirzayanii*, but only at moderate salinity levels, whereas high salinity levels decreased the amount of these compounds [46]. Salt-stressed durum wheat seedlings treated with L-CTS and M-CTS presented a further increase in TPC, TFC and TAA, compared to untreated stressed seedlings. These results indicate that CTS improves salinity tolerance of durum wheat seedlings by improving the accumulation of small antioxidant molecules. In this regard, Picchi and coworkers [47] have recently reported that CTS treatments determine an increase in small antioxidants in durum wheat plants, which results in a mitigation of ozone injury. In particular, the authors observed an activation of the phenylpropanoid pathway after treatment with CTS nanoparticles either unloaded or loaded with the antioxidant compound N-acetyl cysteine, whereas treatment with CTS alone had no effect [47]. In the present study, both TPC and TFC were found to be positively correlated with the DPPH and ABTS radical scavenging activity. This result indicates that phenolic compounds are the dominant component of the non-enzymatic antioxidant system in durum wheat seedlings and that, among phenolic compounds, flavonoids give a significant contribution to the radical scavenging activity under salinity and represent an important target of CTS action. A positive significant correlation between TPC and TAA has been also reported for 112 medicinal plant species [48] and 24 plant species traditionally used for animal health care [49]. In this latter case, the authors did not find significant correlations between the TAA and flavonoids and concluded that, besides flavonoids, there were other phenolic compounds that contributed to the TAA in the plant species analyzed [49]. In regard to durum wheat, there is a large body of literature that specifically focuses on phenolic compounds in the edible seeds and their derived end-products, because of their possible beneficial effects on human health [50,51]. Conversely, the ameliorating effect of phenolic compounds on the phytotoxicity caused by exposure of durum wheat plants to various stressors is still poorly investigated. In this contest, the results obtained in the present study contribute to fill this gap of knowledge by suggesting a role of phenolics in helping durum wheat plants to cope with oxidative stress that arises during exposure to salinity.

Non-enzymatic antioxidants are essential in the protection of cellular components against injuries induced by ROS, but they cannot neutralize reducing radicals, such as superoxide or metastable hydroperoxides [52]. For this reason, nature has evolved antioxidant enzymes able to eliminate superoxide and hydroperoxides. SOD is the first line of defense against ROS and catalyzes the dismutation of the superoxide anion into hydrogen peroxide, which is also toxic and is reduced to water by CAT and POX [19]. Our findings revealed that salinity strongly increases the activities of the antioxidant enzymes SOD and CAT and, to a lesser extent, of total POX. This suggests that, in durum wheat seedlings, CAT and total POX are not equally important in scavenging hydrogen peroxide under salinity. Many studies have demonstrated that the increase in the activity of these enzymes is a common response of plants to salinity, although the relative importance of each type of enzyme (SOD, CAT, POX, etc.) required for ROS scavenging differs from one species to another [53]. The exogenous application of L-CTS and M-CTS further increased CAT activity in salt-stressed shoots, thus confirming the major role played by CAT as hydrogen-peroxide-scavenging enzyme in durum wheat seedlings under salt stress. Similarly, pretreatment with CTS under salt stress resulted in increased activities of the antioxidant enzymes in *Catharanthus roseus* [54], soybean [30], rice [55], safflower and sunflower [56]. Further, in bread wheat exposed to salinity, a significant increase in SOD, CAT and POX activities and a consequent alleviation of the oxidative stress was observed after CTS treatment of seeds [57], leaves [58] or CTS addition to the nutrient solution [33].

Due to the essential role of NADPH in the maintenance of the redox state of the cell, NADP-producing enzymes are considered as antioxidant enzymes that can be included in this group together with SOD, CAT and POX [23]. The role of these enzymes in plant defence against the oxidative stress under salt stress has been widely demonstrated. An increase in the activity NADPH-producing enzymes has been reported in olive [22], Arabidopsis [59] and common bean [60]. Moreover, transgenic Arabidopsis plants overexpressing the rice NADP-ME gene have shown a better growth performance under salinity compared to the wild-type plants [61]. In concordance with previous findings, our results showed an increase in the activity of the G6PDH in durum wheat seedlings exposed to salinity. G6PDH catalyses the rate-limiting step of the pentose phosphate pathway, which provides not only NADPH but also carbon skeletons for the synthesis of phenolic compounds [62]. Therefore, it is feasible that the increased activity of this enzyme may in part explain the parallel increase observed in the TPC and TFC content under salinity. CTS treatment did not result in a further increase of the G6PDH activity, and this suggests that other enzymes involved in the phenylpropanoid pathway could be responsible for the synergetic effect of CTS on TPC accumulation under salinity. 

It is worth noting the observation that, in durum wheat seedlings, the enzymatic and the non-enzymatic antioxidants were found to be positively correlated to each other and with the TAA. This probably reflects a strict relation between the different components of the antioxidant system, which act synergistically to induce a concerted antioxidant response of durum wheat to salinity, a defense response that can be further elicited by exogenous application of CTS. The present study provides a primer for future investigations aimed at investigating further mechanisms of CTS-induced tolerance to salinity in durum wheat. In this regard, the possible effect of CTS on Na^+^ uptake and K^+^ retention merits a detailed investigation, since the maintenance of Na^+^ and K^+^ homeostasis in the shoots has been demonstrated to play a crucial role in the determination of salinity tolerance in durum wheat [9].

## 4. Materials and Methods

### 4.1. Preparation of CTS Solutions

Three types of commercial CTS at low (L-CTS, 50–190 kDa), medium (M-CTS, 190–310 kDa) and high MW (H-CTS, 310–375 kDa) CTS with 75–85% deacetylation were purchased from Sigma-Aldrich (Saint Louis, MO, USA). A one hundred milligrams per liter CTS solution was prepared by dissolving the required amount of powder in 0.5% acetic acid. This concentration was chosen on the basis of a preliminary experiment carried out using different CTS concentrations (10–250 mg/L), which showed that 100 mg/L CTS gave the best growth performance when applied to durum wheat seedlings exposed to 200 mM NaCl stress. The pH of the solution was adjusted to 6.0 by using 1 M NaOH. 

### 4.2. Experimental Design and Treatments

The durum wheat cv. Svevo was used in this study. This genotype was chosen since its genome was recently sequenced and annotated [63] and for this reason it is considered the reference genotype for durum wheat. Seeds were surface sterilized with 5% sodium hypoclorite solution under stirring for 10 min and then were rinsed several times with distilled water. After sterilization, the seeds were transferred to Petri dishes (10 seeds per Petri dish) containing distilled-water-saturated Whatman filter paper and incubated for 3 days in the dark at 25 °C and 85% relative humidity in a growth incubator (Heraeus HPS 1500). Then, 100 plants were grown hydroponically in Hoagland nutrient solution in a growth chamber with a light/dark cycle of 16/8 h at 25/15 °C, 70% relative humidity and a light (250 W Powerstar HQI metal halide lamps, Osram) intensity of 500 μmol (photon) m^−2^ s^−1^. Seedlings were grown under normal growth conditions for 7 days and then they were divided into five groups (20 plants per treatment): (1) control (C), no NaCl addition to the 50 mL Hoagland solution; (2) salt stress (NaCl), 200 mM NaCl addition to the 50 mL Hoagland solution; (3) NaCl + L-CTS, 200 mM NaCl addition to the 50 mL Hoagland solution + foliar spray with 3 mL 100 mg/L L-CTS; (4) NaCl + M-CTS, 200 mM NaCl addition to the 50 mL Hoagland solution + foliar spray with 3 mL 100 mg/L M-CTS; (5) NaCl + H-CTS, 200 mM NaCl addition to the 50 mL Hoagland solution + foliar spray with 3 mL 100 mg/L H-CTS. The growth solution (containing NaCl where required) was renewed every two days. The seedlings were harvested after 7 days after treatments. The experiment was repeated three times and each experiment was used as a biological replicate for all the determinations. Growth parameters were determined on fresh material, whereas the biochemical assays were carried out on the shoot harvested and stored at −80 °C until use.

### 4.3. Determination of Growth Parameters

For the determination of the growth parameters, durum wheat seedlings were cut between the shoot and the root. The shoot height and the primary root length were determined on fresh material by using a meter scale. Dried biomass was determined after drying samples at 70 °C overnight.

### 4.4. Preparation of Shoot Extracts 

For the determination of the superoxide anion production rate and the enzymatic activities, a crude protein extract was prepared essentially as reported in [64]. The shoot tissue was ground under nitrogen, using a pre-chilled mortar and pestle. Five hundred milligrams of the ground powder were resuspended in 10 mL of cold 50 mM sodium phosphate buffer (pH 7.0). After centrifugation of the homogenate at 35,000× *g* at 4 °C for 15 min, the supernatant was collected and its protein content was determined according to [65], using bovine serum albumin as the standard. 

For the determination of TPC, TFC and TAA, the shoot tissue was ground under nitrogen, using a pre-chilled mortar and pestle. Twenty milligrams of the ground powder were resuspended in 2 mL of acidified methanol (methanol:HCl, 99:1 *v*/*v*) and the suspension was ultrasonicated for 30 min. After centrifugation of the suspension at 35,000× *g* at 4 °C for 15 min, the supernatant was collected and used for the determinations.

### 4.5. Determination of ROS Production

The superoxide anion production rate was determined essentially as reported in [66], by following the epinephrine oxidation to adrenochrome. The reaction was started by the addition of 0.2 mg of crude protein extract to the reaction mixture containing 1 mM epinephrine in 2 mL of 50 mM sodium phosphate buffer (pH 7.0) and the adrenochrome generation was measured by following the absorbance increase at 480 nm (ε_480nm_ = 4.00 mM^−1^·cm^−1^). The superoxide anion production rate was expressed as μmol per min/g DW. 

The hydrogen peroxide content was determined according to [67]. The shoot tissue (0.2 g) was homogenized in an ice bath with 3 mL of 0.1% (*w*/*v*) trichloroacetic acid. The homogenate was centrifuged at 12,000× *g* for 15 min and 0.5 mL of the supernatant was added to 0.5 mL of 10 mM potassium phosphate buffer (pH 7.0) and 1 mL of 1 mM KI. The absorbance of the supernatant was measured at 390 nm. The hydrogen peroxide content was obtained using a standard curve and expressed as μmol/g DW.

### 4.6. Determination of MDA Content

The MDA content was determined using a thiobarbituric acid reaction essentially as reported in [66]. Briefly, the shoot tissue (0.5 g) was homogenized in 10 mL of 5% trichloroacetic acid and the homogenate was centrifuged at 12,000× *g* for 15 min. Then, 5 mL of 0.5% thiobarbituric acid were added to 2 mL of supernatant; the mixture was heated at 100 °C for 15 min and cooled on ice immediately afterwards to stop the reaction. The absorbance was recorded at 450, 532 and 600 nm. The MDA content was expressed as μmol/g DW.

### 4.7. Determination of TPC and TFC

TPC was determined using the Folin–Ciocalteu assay according to [68]. Briefly, 200 μL extract (2 mg/mL) were mixed with 900 μL of Folin–Ciocalteau reagent diluted 1:10 and 900 μL of 7% Na_2_CO_3_. The solution was incubated at room temperature in the dark for 1 h and then its absorbance at 750 nm was measured. TPC was expressed as mg of ferulic acid equivalents (FE)/g DW.

TFC was determined following the method described by [69], with minor modifications. Briefly, 250 μL of extract (4 mg/mL) were mixed with 1 mL of distilled water and 80 μL of 5% NaNO_2_. Five minutes later, 150 μL of 10% AlCl_3_ were added to the mixture. Then, 500 μL of 1 M NaOH were added after 6 min and the absorbance at 415 nm was measured. TFC was expressed as mg of catechin equivalents (CE)/g DW.

### 4.8. Determination of TAA

TAA of the extract was determined using the DPPH assay and the ABTS assay, as described by [70], with minor modifications. For the DPPH assay, a DPPH radical solution having an absorbance value of 0.75–0.80 at 525 nm was prepared daily by dissolving 5 mg of DPPH radical in 100 mL of methanol/water mixture (50:50, *v*/*v*). For the evaluation of the DPPH radical scavenging activity, 100 mL of extract (10 mg/mL) were mixed with 4.9 mL of DPPH radical solution. The mixture was incubated at room temperature in the dark for 30 min. After incubation, the absorbance at 525 nm was measured. For the ABTS assay, an ABTS radical solution having an absorbance value of 0.75–0.80 at 734 nm was prepared by dissolving 5 mg of ABTS and 1 mg of K_2_S_2_O_8_ in 100 mL of water. For the evaluation of the ABTS radical scavenging activity, 100 mL of extract (10 mg/mL) were mixed with 4.9 mL of ABTS radical solution. The mixture was incubated at room temperature for 30 min and then the absorbance at 734 nm was measured. For both the assays, TAA activity was expressed as μmol Trolox equivalent (TE)/g DW.

### 4.9. Determination of the Enzymatic Activities

SOD activity was determined as reported by [71], by using a cytochrome c method and xanthine/xanthine oxidase as the source of superoxide radicals. Xanthine oxidase (0.02 enzymatic units, E.U.) was added to a reaction mixture containing 100 μM xanthine and 20 μM cytochrome c in 2 mL of 50 mM sodium phosphate buffer (pH 7.0) and the formation of reduced cytochrome c was monitored by following the absorbance increase at 550 nm (ε_550nm_ = 21 mM^−1^·cm^−1^). After 2 min, the crude protein extract (0.05 mg) was added to the reaction mixture and the percentage of inhibition of the rate of cytochrome c reduction was used to estimate SOD activity. One E.U. of SOD was defined as the amount of enzyme necessary to determine 50% inhibition in the rate of cytochrome c reduction at 25 °C.

CAT activity was monitored oxygraphically according to [72]. The crude protein extract (0.005 mg) was added to a reaction mixture containing 0.06% hydrogen peroxide in 2 mL of 50 mM sodium phosphate buffer (pH 7.0) and the enzyme activity was evaluated by monitoring the amount of evolved oxygen because of hydrogen peroxide decomposition. One E.U. of CAT was defined as the amount of enzyme that produced 1 μmol oxygen/min at 25 °C.

Total POX activity was determined as reported by [73]. The crude protein extract (0.05 mg) was added to a reaction mixture containing 10 mM guaiacol and 100 μM hydrogen peroxide in 2 mL of 50 mM sodium phosphate buffer (pH 7.0) and the oxidation of guaiacol was monitored by following the absorbance increase at 470 nm (ε_470nm_ = 26.6 mM^−1^·cm^−1^). One E.U. of total POX was defined as the amount of enzyme that caused the oxidation of 1 μmol guaiacol/min at 25 °C.

G6PDH activity was determined as reported by [74]. The crude protein extract (0.25 mg) was added to a reaction medium containing 5 E.U. hexokinase, 2.5 mM glucose, 500 μM ATP and 400 μM NADP^+^ in 2 mL of 50 mM sodium phosphate buffer (pH 7.0) and the NADPH generation was monitored by following the absorbance increase at 340 nm (ε_340nm_ = 6.22 mM^−1^·cm^−1^). One E.U. of G6PDH was defined as the amount of enzyme that reduced 1 μmol NADP^+^/min at 25 °C.

NADP-ME activity was determined as reported by [75]. The crude protein extract (0.25 mg) was added to a reaction medium containing 10 mM malate and 500 μM NADP^+^ in 2 mL of 50 mM sodium phosphate buffer (pH 7.0) and the NADP^+^ reduction was monitored by following the absorbance increase at 340 nm. One E.U. of NADP-ME was defined as the amount of enzyme that reduced 1 μmol NADP^+^/min at 25 °C.

All the specific enzyme activities were expressed as E.U./g DW.

### 4.10. Statistical Analysis

One-way analysis of variance (ANOVA) was carried out with respect to each of the traits investigated. The results were expressed as mean of three independent experiments ± SD. Significant differences among the means were determined using the Tukey’s multiple range test (*p* ≤ 0.05). Relationship between variables were determined using Pearson correlation coefficients (*p* ≤ 0.05) and the PCA based on correlation was carried out on the entire dataset of the shoot. The statistical analysis was performed using the JMP software (version 8.0, SAS Institute Inc., Cary, NC, USA).

## 5. Conclusions

In conclusion, our study confirms that salt stress significantly induces adaptive defense mechanisms in plants, such as the induction of enzymatic and non-enzymatic antioxidants. Durum wheat seedlings responded to salt stress by increasing TPC, TFC, SOD, CAT, total POX and G6PDH. Moreover, our results indicate that CTS is an effective biostimulator that exerts a positive effect on plant growth. The application of L-CTS and M-CTS significantly reduced the salt-induced oxidative damages in durum wheat seedlings by further enhancing the accumulation of phenolic compounds and the activation of the antioxidant enzyme CAT. Thus, these findings suggest that the application of CTS may represent a sustainable approach to counteract the detrimental effect of salinity. Since its effects vary with the MW, the oligomer size required for biological activity should be defined before application to the crop. Finally, it is noteworthy that the availability of the whole genome sequence of the cv. Svevo is of great utility for future studies on the molecular mechanisms underlying the durum wheat response to salinity and CTS treatments. The results obtained could provide useful information for the breeding programs aimed at the selection of new salt-tolerant, high-yielding durum wheat genotypes.

## Figures and Tables

**Table 1:** Growth parameters of durum wheat seedlings grown under control, NaCl stress and NaCl stress + CTS treatment.

Treatment	Shoot		Root	
	Length (cm)	Dried Biomass (mg)	Length (cm)	Dried Biomass (mg)
Control	30.37 ± 1.08 ^a^	57.26 ± 1.26 ^a^	18.68 ± 1.61 ^a^	26.89 ± 0.96 ^a^
NaCl	19.52 ± 1.16 ^d^	35.57 ± 1.09 ^e^	7.38 ± 1.52 ^d^	10.89 ± 1.28 ^d^
NaCl + L-CTS	25.27 ± 0.85 ^b^	46.89 ± 1.34 ^c^	13.31 ± 1.38 ^b^	20.39 ± 1.34 ^b^
NaCl + M-CTS	26.59 ± 1.46 ^b^	51.23 ± 1.46 ^b^	14.22 ± 1.42 ^b^	17.88 ± 1.03 ^c^
NaCl + H-CTS	21.44 ± 1.67 ^c^	39.49 ± 1.26 ^d^	10.49 ± 1.14 ^c^	16.84 ± 1.13 ^c^

Data are reported as mean ± S.D. (*n* = 3). Different uppercase letters indicate significant differences according to the Tukey’s test (*p* ≤ 0.05).

**Table 2:** Total phenolic content (TPC), total flavonoid content (TFC) and total antioxidant activity (TAA) in the shoot of durum wheat seedlings grown under control, NaCl stress and NaCl stress + CTS treatment.

Treatment	TPC (mg FE g^−1^ DW)	TFC (mg CE g^−^^1^ DW)	TAA (μmol TE g^−^^1^ DW)	
			DPPH	ABTS
Control	9.28 ± 0.45 ^d^	3.87 ± 0.25 ^c^	132.3 ± 1.0 ^c^	100.3 ± 2.6 ^c^
NaCl	15.52 ± 0.37 ^c^	5.85 ± 0.29 ^b^	163.8 ± 3.5 ^b^	132.4 ± 0.1 ^b^
NaCl + L-CTS	19.08 ± 0.24 ^a^	6.99 ± 0.32 ^a^	181.2 ± 0.8 ^a^	140.1 ± 0.8 ^a^
NaCl + M-CTS	17.73 ± 0.49 ^b^	6.55 ± 0.33 ^ab^	174.9 ± 0.8 ^a^	134.9 ± 1.2 ^ab^
NaCl + H-CTS	16.88 ± 0.20 ^b^	6.33 ± 0.12 ^ab^	172.9 ± 1.3 ^ab^	129.2 ± 1.1 ^b^

Data are reported as mean ± S.D. (*n* = 3). Different uppercase letters indicate significant differences according to the Tukey’s test (*p* ≤ 0.05).
